# Supplementary material for: Marine Actinomycetes-Derived Secondary Metabolites Overcome TRAIL-Resistance via the Intrinsic Pathway through Downregulation of Survivin and XIAP
Source: Cells. 2020 Jul 22;9(8):1760. doi: 10.3390/cells9081760 (PMC7464567; doi:10.3390/cells9081760)
Supplement: Supplementary file 1 [file cells-09-01760-s001.pdf]

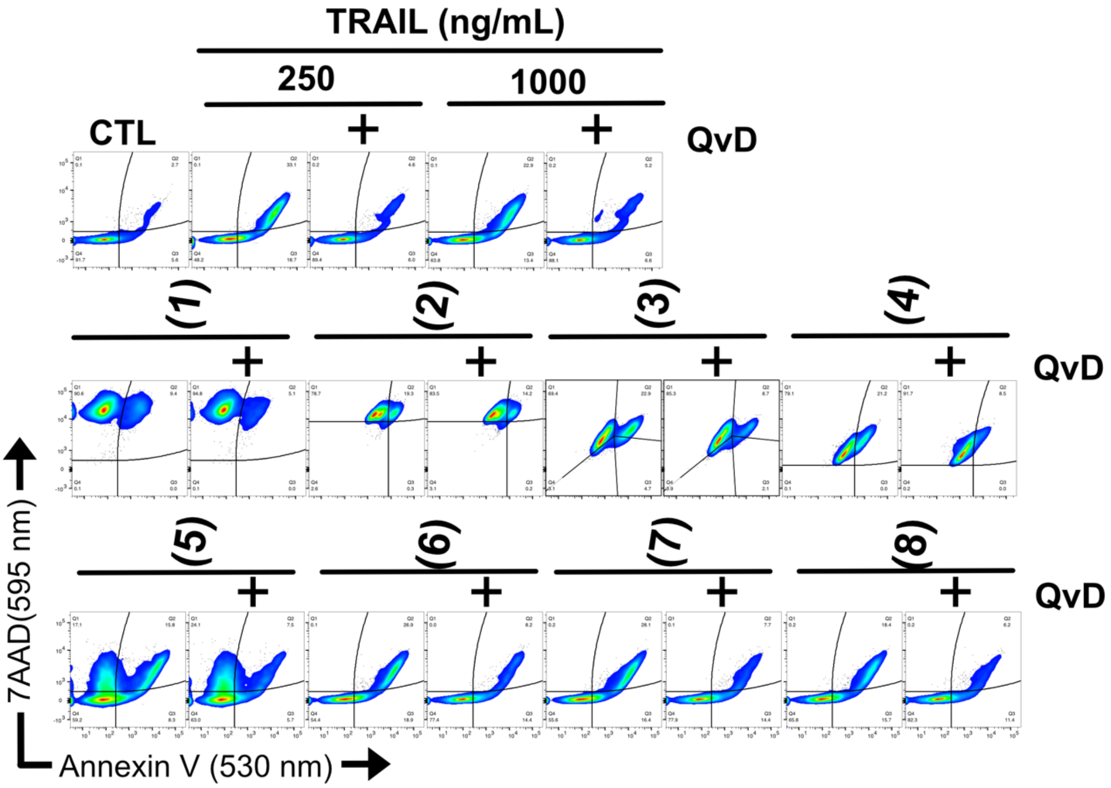

Figure S1: LEGEND

Flow cytometric analysis monitoring the effect of *pan*-caspase inhibitor (QvD) on marine compound-induced apoptosis in HCT116 colon cancer cell line. Cells were pre-incubated for 30 min with and without *pan*-caspase inhibitor QvD (10 µM) then treated with marine compounds (50 µM) or TRAIL (0.25 and 1 µg/mL) for 24 h. following incubation, cells were stained with 7AAD and Annexin V and fluorescence was analyzed by flow cytometry.

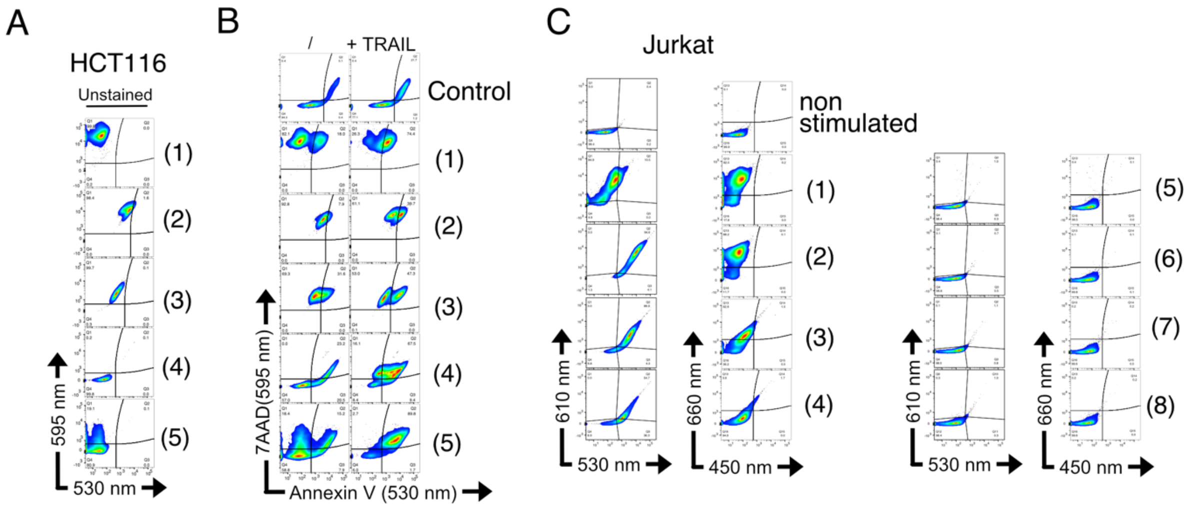

Figure S2: Autofluorescence screening and monitoring apoptosis-induced by marine compounds in HCT116 and Jurkat cells using flow cytometry. (A &B) HCT116 cells were incubated with the depicted compounds for 24h and stained (A) or not (B) with 7AAD and Annexin V and fluorescence was analyzed by flow cytometry. (C) Jurkat cells were treated as above and compound-induced autofluorescence was assessed by flow cytometry at 450, 530, 610 and 660 nm**.**

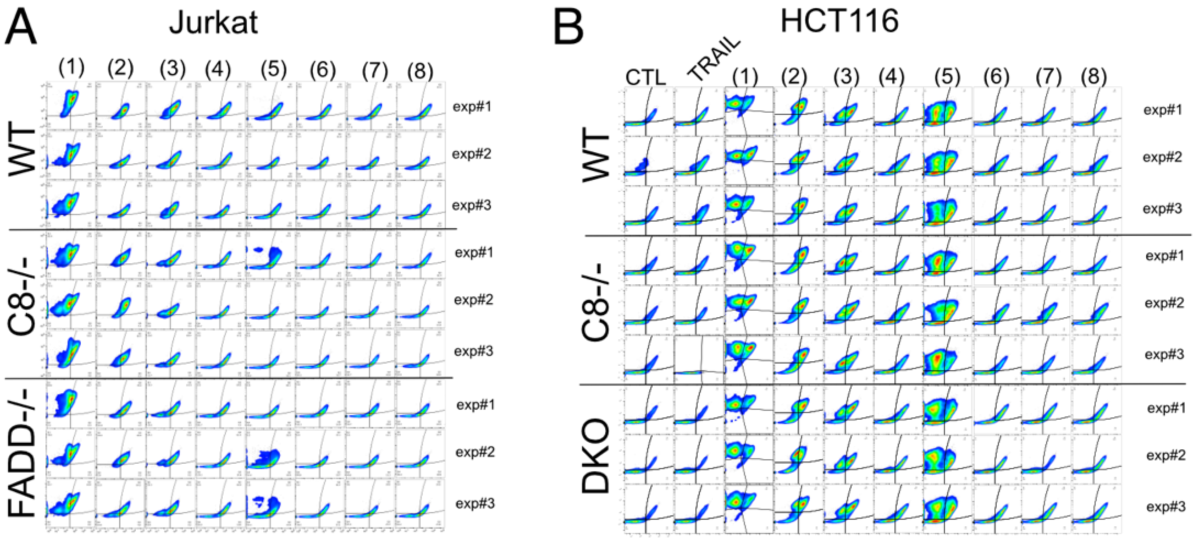

Figure S3: Detection of apoptosis induced by marine compounds in HCT116 and Jurkat isogenic cells. (A) HCT116 (WT), Cas8-deficient HCT116-Cas8-/, and TRAIL receptors deficient HCT116-TRAIL-R1/TRAILR2-/- (B) Jurkat WT, Jurkat-Caspase-8-/-, and Jurkat-FADD-/-. Isogenic cells were incubated with the compounds for 24 h and then subjected to dual staining with annexin V/7AAD/. Apoptosis rate was determined by flow cytometry.

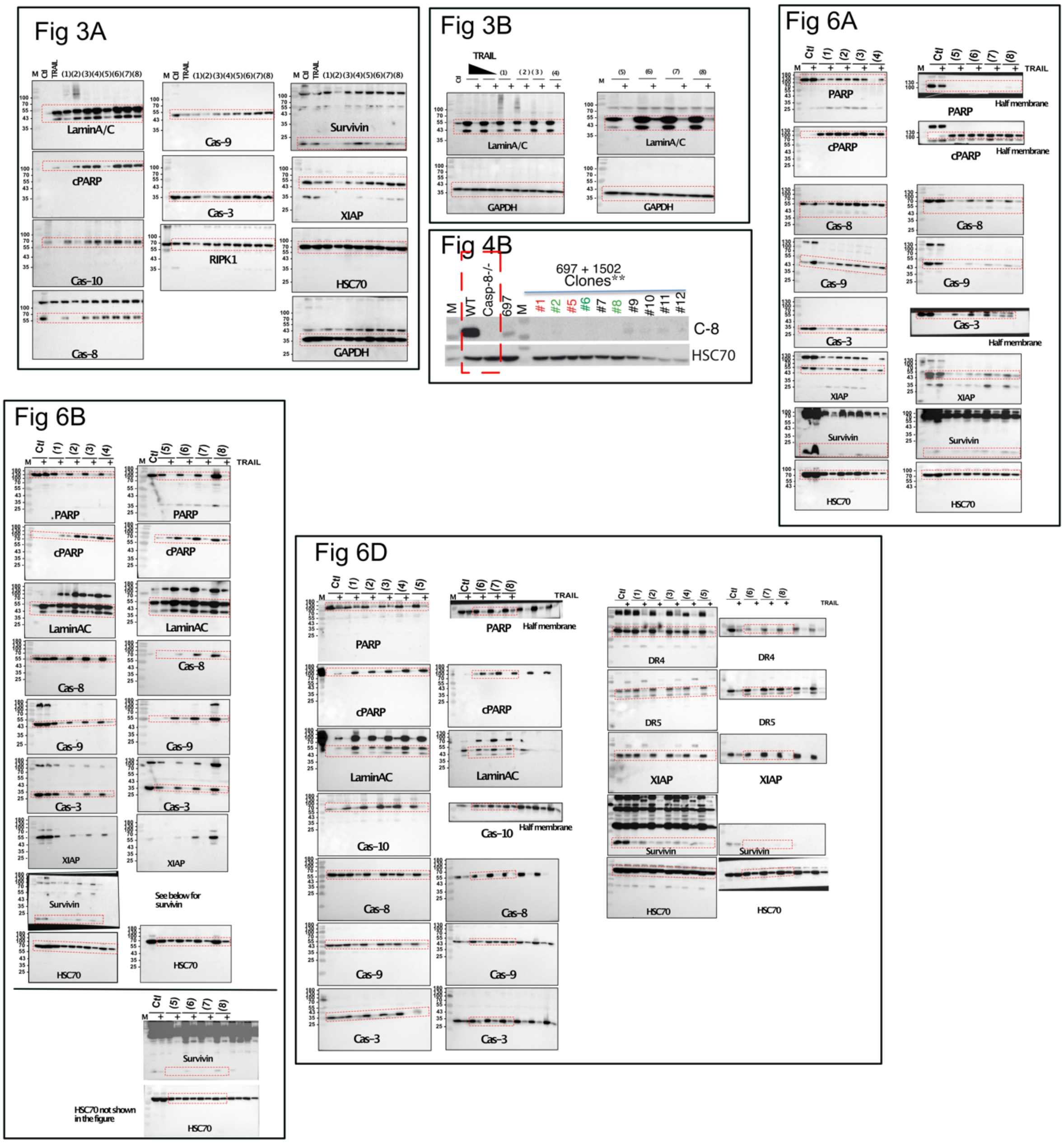

Figure S4: Raw data of the Western blots
